# Chemical Profiling, Antioxidant, Anticholinesterase, and Antiprotozoal Potentials of *Artemisia copa* Phil. (Asteraceae)

**DOI:** 10.3389/fphar.2020.594174

**Published:** 2020-12-04

**Authors:** María José Larrazábal-Fuentes, Carlos Fernández-Galleguillos, Jenifer Palma-Ramírez, Javier Romero-Parra, Kevin Sepúlveda, Alexandra Galetovic, Jorge González, Adrián Paredes, Jorge Bórquez, Mario J. Simirgiotis, Javier Echeverría

**Affiliations:** ^1^ Unidad Alimentos, Departamento de Ciencias de los Alimentos y Nutrición, Facultad de Ciencias de la Salud, Universidad de Antofagasta, Antofagasta, Chile; ^2^ Instituto de Farmacia, Facultad de Ciencias, Universidad Austral de Chile, Valdivia, Chile; ^3^ Departamento de Química Orgánica y Fisicoquímica, Facultad de Ciencias Químicas y Farmacéuticas, Universidad de Chile, Santiago, Chile; ^4^ Unidad de Parasitología Molecular, Departamento de Tecnología Médica, Facultad de Ciencias de la Salud, Universidad de Antofagasta, Antofagasta, Chile; ^5^ Departamento Biomédico, Universidad de Antofagasta, Antofagasta, Chile; ^6^ Laboratorio de Productos Naturales, Departamento de Química, Facultad de Ciencias Básicas, Universidad de Antofagasta, Antofagasta, Chile; ^7^ Departamento de Ciencias del Ambiente, Facultad de Química y Biología, Universidad de Santiago de Chile, Santiago, Chile

**Keywords:** *Artemisia copa*, Asteraceae, traditional medicine, HPLC-MS, anti-*Trypanosoma cruzi*, Anti-*Trypanosoma* activity, cholinesterase inhibition

## Abstract

*Artemisia copa* Phil. (Asteraceae) (known as copa-copa) is a native species of Chile used as an infusion in traditional medicine by Atacameños people in the Altiplano, highlands of northern Chile. In this research, we have investigated for the first time the cholinesterase inhibition potential against acetylcholinesterase (AChE) and butyrylcholinesterase (BChE), and the chemical profiling of the infusions prepared from the aerial parts of *A. copa* by high resolution spectrometry. In addition, total phenolic, total flavonoid content, antioxidant (DPPH, FRAP, and ORAC) and antiprozoal activity were tested. *Artemisia copa* showed good inhibitory activity against AChE and BChE (3.92 ± 0.08 µg/ml and 44.13 ± 0.10 µg/ml). The infusion displayed a total phenolics content of 155.6 ± 2.9 mg of gallic acid equivalents/g and total flavonoid content of 5.5 ± 0.2 mg quercetin equivalents/g. Additionally, trypanocidal activity against *Trypanosoma cruzi* was found (LD_50_ of 131.8 µg/ml). Forty-seven metabolites were detected in the infusion of *A. copa* including several phenolic acids and flavonoids which were rapidly identified using ultrahigh performance liquid chromatography orbitrap mass spectrometry analysis (UHPLC-Orbitrap-MS) for chemical profiling. The major compounds identified in the infusions were studied by molecular docking against AChE and BChE. The UHPLC-MS fingerprints generated can be also used for the authentication of these endemic species. These findings reveal that *A. copa* infusions can be used as beverages with protective effects.

## Introduction

**FIGURE 1:**
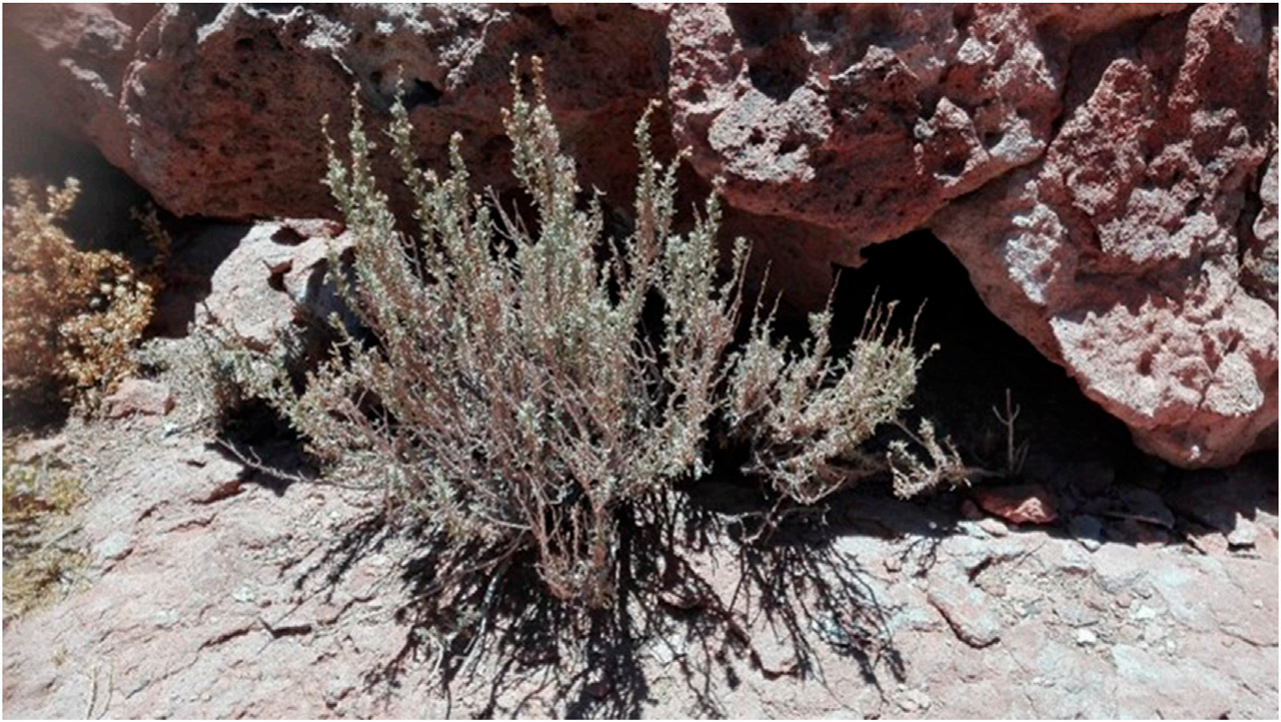
*Artemisia copa* Phil (Asteraceae).

The Andean Altiplano is a South American high plateau located at about 3,700 m above sea level shared by Bolivia, Argentina, part of southern Peru, and Chile. These locations have ecological and environmental importance, and several ancient civilizations arose there, which uses the special fauna and flora as food and medicines for centuries. *Artemisia copa* Phil. (Asteraceae) also knows as “copa-copa” is a Chilean altiplano plant characterized by having small green leaves (Figure 1) and used as an infusion in popular medicine (Giberti, 1983) in the Atacama Desert, North West of Argentina and Altiplano in Northern Chile. It grows between 3,500 and 4,250 m above sea level.

The *A. copa* infusion is used in traditional medicine for the treatment of colds, pneumonia, hypertension, pain, for gall bladder problems, as digestive, often combined with sodium bicarbonate. The infusion mixed with milk is used for stomach pain, cold, and colic. In the form of baths or incense, it serves to combat “mal de aire” and toothache. As a bath, it serves for bone pain. People attribute sedative properties to the species the ability to cause vivid dreams (Giberti, 1983; Montes and Wilkomirsky, 1985; De Nucci, 1988; Paniagua-Zambrana et al., 2020).

Different compounds have been identified in the extracts of aerial parts of *Artemisia copa*. In the dichloromethane and ethanolic extracts have been isolated the flavonoids spinacetin, jaceosidin, axillarin, penduletin, tricin, chrysoeriol (Moscatelli et al., 2006), chrysosplenetin (Morales et al., 2003), jaceidin (de la Fuente et al., 1986), 7-methyljaceidin, kaempherol-6-methyleter-3-*O*-rhamnoglucosyde, and luteolin (Catalán et al., 2007). The guaianolides achillin and deacetylmatricarin have been isolated (de la Fuente et al., 1986). The coumarin scopoletin and phenolic acid *p*-coumaric acid have also been identified (Moscatelli et al., 2006). The essential oils of leaves, twigs, and flowers have been identified monoterpenoids as chrysanthenone, chrysanthenyl acetate, β-thujone, γ-terpinene, *cis*-2-menthenol, limonene and α-pinene, sesquiterpenoids as chamazulene, linalool and linalyl acetate, and homoterpene and other miscellaneous compounds as butyric acid (Catalan et al., 1982; Arze et al., 2004; Catalán et al., 2007; Larrazabal-Fuentes et al., 2019).

*A. copa* has been shown to possess significant biological activities such as antioxidant (Pérez-García et al., 2001; Catalán et al., 2007), analgesic and topical anti-inflammatory activities via inhibition of nitric oxide production, reduction of prostaglandin E2 levels and inhibition of cyclooxygenase-2 activity (Miño et al., 2004), inhibition of synovial phospholipase A2 activity (Moscatelli et al., 2006), spasmolytic activity (Gorzalczany et al., 2013a) and vasorelaxant and hypotensive effects through the inhibition of Ca^2+^ influx via membranous calcium channels and intracellular stores (Gorzalczany et al., 2013b).

Numerous results are reporting on the anti-trypanosomal activity the genus *Artemisia*, artemisinin, and its derivatives and other phytochemicals from *Artemisia* species (Sen et al., 2007; Aloui et al., 2016). However, more intensive research is required to explore the full potential of diverse *Artemisia* species and their chemical ingredients for the eradication of trypanosomal infections (Naß and Efferth, 2018).

Until now, no studies have been reported on the antiprotozoal and enzyme inhibitory properties against cholinesterase of *A. copa*. In the same way, no metabolomic analyzes of the chemical compounds present in *A. copa* have been reported. Ultra-high-performance liquid chromatography (UHPLC) coupled with high-resolution mass spectrometry (HR-MS) is a modern and fast technique that allows the identification of accurate mass analysis. Moreover, is today a key tool for plant chemotaxonomy or metabolic profiling of plant samples (Simirgiotis et al., 2016b; Echiburu-Chau et al., 2017; Areche et al., 2020; Rodriguez et al., 2020).

Since herbal native infusions are receiving increasing attention for the number of physiological benefits they can bring to human health (Coelho et al., 2016) and as part of our continuing research of medicinal plants from Atacama Desert (Echiburu-Chau et al., 2017; Ardiles et al., 2018; Areche et al., 2020; Rodriguez et al., 2020) and native plants with potentiality as cholinesterase inhibitors (Barrientos et al., 2020a; Barrientos et al., 2020b), we investigated the infusions of the medicinal plant *A. copa* using screening tests to evaluate their antioxidant, antiprotozoal and enzyme inhibitory properties against cholinesterase and high-resolution UHPLC-MS/MS for the chemical profiling for the first time. Additionally, molecular docking of the major compounds was investigated.

## Materials and Methods

### Chemicals and Regents

Solvents type UHPLC-MS, formic acid type LC-MS, and chloroform were from Merck (Santiago, Chile). Ultrapure water was obtained from a water purification system brand Millipore (Milli-Q Merck Millipore, Chile). Flavonol standards, (catechin, isoflavones, and flavonoids, all standards with a high purity: 95% by HPLC) were acquired from ChromaDex (Santa Ana, CA, United States), Sigma Aldrich (Saint Louis, Mo, United States), or Extrasynthèse (Genay, France). Folin-Ciocalteu reagent, NaOH, Na_2_CO_3_, AlCl_3_, FeCl_3_, HCl, NaNO_2_, trichloroacetic acid, quercetin, 6-hydroxy-2,5,7,8-tetramethylchromane-2-carboxylic acid (Trolox), sodium acetate, Gallic acid, TPTZ, nitroblue tetrazolium, DPPH (1,1-diphenyl-2-picrylhydrazyl radical), acetylcholinesterase (AChE), butyrylcholinesterase (BChE), 2,2’-Azobis (2-amidinopropane) dihydrochloride (AAPH), were acquired from Sigma-Aldrich Chemical Company (Santiago, Chile).

### Plant Material

*Artemisia copa* Phil. (Asteraceae) was collected in El Tatio, Antofagasta, Región de Antofagasta, Chile in November 2016. Voucher specimens for the herbarium are kept at the Laboratory of Natural Products of the Universidad de Antofagasta under the number: AC20161115.

### Preparation of the Infusion of *A. copa*

The infusion was prepared using 3 g of dried milled aerial parts (stems and leaves) with 250 ml of boiling deionized water for 30 min. The infusion was filtered (Whatman 1), lyophilized (Labconco 2.5 L), and refrigerated until use at 4^°^C (the yield of the aqueous extraction was 8.6%).

### Ultra-High-Performance Liquid Chromatography-Photodiode Array Detection-Orbitrap Technology-Mass Spectrometry Instrument

A UHPLC high-resolution MS Dionex Thermo Scientific Ultimate 3000 system connected with a Thermo Q-Exactive focus machine was used. For the analysis, 2 mg per ml of lyophilized material was dissolved in methanol-distilled water (1:1 v/v) and 10 µl of filtered solution (PTFE filter) was injected in the instrument, with the specifications set as already informed (Simirgiotis et al., 2016a).

### LC Parameters and Mass Spectrometry Parameters

Liquid chromatography was run using an Acclaim UHPLC C18 column (150 × 4.6 mm ID, 2.5 µm, Thermo Fisher Scientific, Bremen, Germany) set at 25°C. The wavelength detection was 354, 254, 280, and 330 nm, and PDA was acquired from 200 to 800 nm for full characterization of peaks. Mobile phases employed were acetonitrile (B) and 1% formic aqueous solution (A) while the gradient program was: (time in minutes and % B), (0.00 min, 5% B); (5.00 min, 5% B); (10.00 min, 30% B); (15.00 min, 30% B); (20.00 min, 70% B); (25.00 min, 70% B); (35.00 min, 5% B) and 12 min for column equilibration before injections. The flow rate employed was 1.00 ml min^−1^, and the injection volume was 10 µl. Standards dissolved in methanol and infusions were maintained at 10°C during storage in the auto-sampler. The HESI II and OT spectrometer parameters were set as informed previously (Simirgiotis et al., 2016c).

### HPLC-Photodiode Array Detection Quantitation of Main Phenolic Compounds in *A. copa*

Some phenolic compounds were quantified using the UHPLC Dionex 3000 RS with photodiode array detector at flavonoid (255 and 265 nm) and phenolic acid (330 nm) wavelength as reported previously carried out according to ICH Guideline Q2 (R1) (Barrientos et al., 2020b). Some curves for representative compounds, each one covering six points from 0.01 to 0.5 mg/ml solutions, in triplicate, and injecting 10 ml. The linearity of the chromatographic method developed was confirmed by the coefficients of determination all around 0.999. For kaempferol based compounds the calibration curve was performed with kaempferol standard, (*R* ^2^ = 0.9997) and for quercetin-based compounds, the curve was performed with quercetin standard (*R* ^2^ = 0.9989), and other phenolic acid were quantified using a chlorogenic acid curve (*R* ^2^ = 0.9994). Using PDA, the lowest detection (LOD) and quantification (LOQ) limits, 0.0115 and 0.0322 mg/L respectively, were obtained for kaempferol, while the highest limits, 0.5543, and 1.476 mg/l were for chlorogenic acid. For the quantification the prepared infusion was injected (10 ml) in triplicate at 25°C.

### Determination of Total Phenolic and Flavonoid Contents

The analyses of total phenolic compounds and flavonoid content were performed following a previous methodology of Folin-Ciocalteu (Morales and Paredes, 2014) and the aluminum chloride method (Simirgiotis et al., 2013) in microplate with some modifications. For total phenolic, a standard solution of gallic acid serial dilution (25–300 µg/ml in 10% EtOH) was used to prepare a calibration curve. Results were expressed as gallic acid equivalent (GAE)/g of dry plant. Flavonoid content was calculated using a quercetin standard calibration curve (2.5–250 μg/ml in ethanol) and the results were expressed as mg quercetin equivalents (QE)/g of dry plant. The absorbances for each methodology was determined using a microplate reader Synergy TM HT Multi-Mode.

### Antioxidant Assays

#### 1,1-Diphenyl-2-Picrylhydrazyl Radical Free Radical Scavenging Activity

The DPPH• (3.9 ml, 0.075 mM, in methanol) method reported by (Zeraik et al., 2016) was used to evaluate the antioxidant activity of *A. copa*. Briefly, 400 µl of a solution, (2 mg/ml for standards), plus 2 ml of DPPH solution were adjusted with the solvent methanol to an absorbance of 1.10 ± 0.02 at 517 nm. The mixture was homogenized using a vortex, allowed to react in the dark at room temperature for 20 min, after which time absorbance was measured at 517 nm. Gallic acid was used as a reference standard. The results were expressed as IC_50_ (concentration of infusion or standard in μg/ml required to inhibit 50% of DPPH radical present in solution).

#### Ferric Reduction Ability-Antioxidant Power Test

For the Ferric Reducing Antioxidant Power (FRAP) test, the methodology determined by (Benzie and Strain, 1996) was performed with slight modifications (Morales and Paredes, 2014). The fresh working solution (FRAP reagent) included 300 mM acetate buffer (3.1 g C_2_H_3_NaO_2_^.^3 H_2_O and 16 ml CH_3_COOH pH 3.6), 10 mM TPTZ (2,4.6-tripyridyl-s-triazine) solution in 40 mM HCl, and 20 mM FeCl_3_^.^6H_2_O solution were prepared. Trolox was used as standard solution. Briefly, to some 100 µl of the solution (or standards at 1 mg/ml), 3 ml of the FRAP solution was added and mixed using a vortex, allowed to react in the dark at room temperature for 5 min. The absorbance measurement of the colored Fe-TPTZ complex was performed at 593 nm. Absorbance values were replaced in the Trolox standard curve equation (μmol/l). The results were expressed as mg equivalents of Trolox/100 g of the dry plant.

#### Oxygen Radical Absorbance Capacity

The Oxygen radical absorbance capacity (ORAC) activity was determined according to the method previously reported by Huang with some modifications (Huang et al., 2002). Fluorescein (3′,6′-dihydroxyspiro[2-benzofuran-3,9′-xanthene]-1-one) stock solution (4 μM) was made in 75 mM phosphate buffer pH 7.4, stored at 4°C and used as the fluorescent probe (Zhang et al., 2009). AAPH (203.4 mg) was freshly prepared in 15 ml of 75 mM phosphate buffer and used to generate the peroxyl radical. Trolox was used as internal standard. In all the experiments, 150 µl of fluorescein was added to each well. Blank wells received 25 μl of phosphate buffer, standards 25 μl of Trolox dilution, and samples 25 μl of the sample. Excitation was monitored at 485 nm and emission at 528 nm with a 20 nm bandpass on an HTX Multi-Mode Microplate Reader. The results were obtained by quadratic regression equation (Trolox/samples vs. fluorescence decay curves) and expressed as μM Trolox equivalents per 100 g of the dry plant.

### Cholinesterase (ChE) Inhibition

The infusions were evaluated by the Ellman’s method, with some modifications (Ellman et al., 1961). Sample solution (50 µl, 2 mg/ml) was mixed with 120 µl DTNB (0.3 mM, 5,5-dithio-bis(2-nitrobenzoic) acid) and AChE (0.026 U/ml, AChE from Electric eel), or BChE (0.026 U/ml, BChE from horse serum) solution (25 µl) in Tris-HCl buffer 50 mM (pH 8.0) in a 96-well microplate and incubated for 20 min at 37°C. The reaction was then initiated with the addition of 20 µl of ATCI (1.5 mM, acetylthiocholine iodide) or BTCl (1.5 mM, butyrylthiocholine chloride). A blank was prepared for all reaction reagents without enzymes solution. The absorbances were recorded at 405 nm for 30 min at 37^o^C and the results were expressed as IC_50_ values (concentration range from 0.05 to 25 µg/ml).

### Molecular Docking Studies

The geometries and partial charges of major flavonoids apigenin-7-*O*-glucoside, kaempferol-3-*O*-galactoside, kaempferol-3-*O*-acetyl-glucoside and kaempferol-7-rhamnoside contained in *A. copa* infusions, as well as the known cholinesterase inhibitor galantamine, was fully optimized using the DFT method with the standard basis set PBE0/ 6-311 + g* (Adamo and Barone, 1999). Molecular docking simulations over *Tc*AChE and *h*BChE were performed using Autodock 4.2. All calculations were performed in Gaussian 09W software.

### Parasites and Cell Cultures

*T. cruzi* (CL strain expressing beta-galactosidase) was used throughout this study (Andriani et al., 2011). Vero cells were infected with Tissue culture-derived trypomastigotes as previously described (San Francisco et al., 2017). Briefly, Vero cell monolayers were cultured in RPMI-1640 media supplemented with 10% fetal bovine sera. Culture flasks of 75 cm^2^ (Nunc, Eas YFlasks, Thermo Scientific, Bremen, Germany) containing subconfluent cultures of Vero cells, were infected with Tissue culture-derived trypomastigotes (CL strain) in a parasite/cell ratio equal to 5:1. After 3 h of infection, cell cultures were washed with PBS to remove parasites that did not penetrate into the cell and cultures were re-incubated in RPMI-1640 medium at 37°C in a humidified 5% CO_2_ atmosphere. Five days later, a pure population of *T. cruzi* trypomastigotes released in cell culture supernatants were harvested by centrifugation. *L. amazonensis* (IFLA/BR/67/PH8) was propagated as promastigotes at 26°C in M199 media supplemented with 5% penicillin/streptomycin, 0.1% hemin (25 mg/ml in 0.1N NaOH), 10 mM adenine and 10% FBS, pH 7.5 as previously described (Sauter et al., 2019). Antiprotozoal activity against *T. cruzi* was evaluated as previously described (Gutiérrez et al., 2013). Briefly, serial dilutions of each infusion were performed in 96 wells microplates, in the range of 1000 to 0.48 μg/ml in a final volume of 100 µl. Then, 100 µl of a suspension of trypomastigotes 1 × 10^7^ ml^−1^ were added and microplates were incubated 48 h. Finally, a substrate, the β-D-galactopyranoside was added, and the mixture incubated 4 h and finally, the enzymatic activity was recorded at 570 nm in a Tecan Infinite M200 Pro (Tecan, Männedorf, Switzerland). Leishmanicidal activity was evaluated as described (Loyola et al., 2001). Briefly, serial dilutions of each infusion were performed as described above. Then, 100 µl of a suspension of 1 × 10^7^ parasites ml^−1^ of *L. amazonensis* promastigotes were added and the microplates incubated 48 h. Finally, the number of live parasites were count in a Neubauer chamber.

### Statistical Analysis

The results are expressed as mean values ± standard error using GraphPad Prism 8 (GraphPad software Corporation, La Jolla, CA, United States). One-way analysis of variance (ANOVA; repeating three times each measurement of sample solutions) (*p* < 0.05) was used for comparison of means.

## Results and Discussion

### Chemical Profiling

**FIGURE 2:**
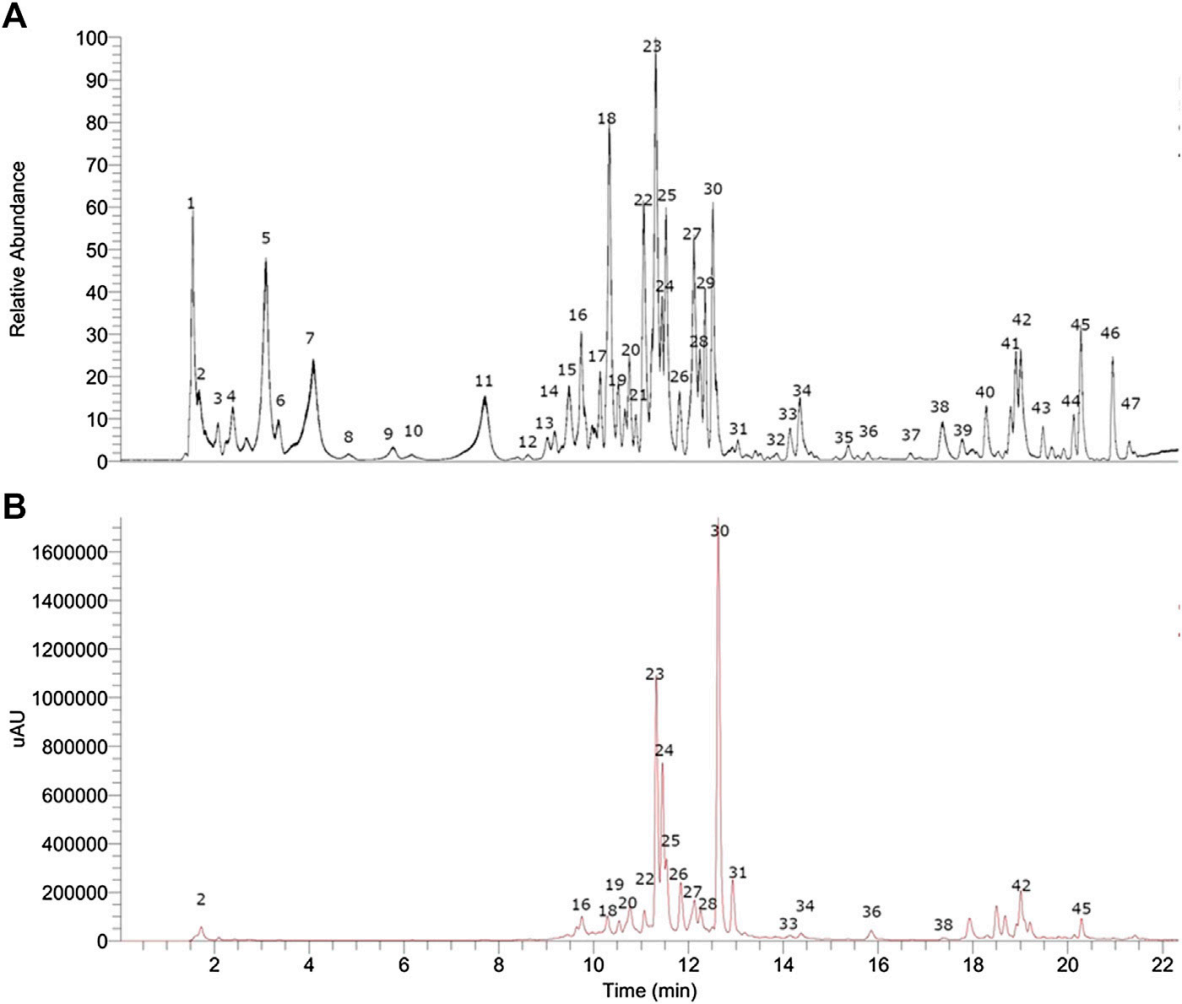
UHPLC chromatograms of *Artemisia copa* Phil. **(A)** TIC chromatogram, **(B)** UV at 280 nm chomatogram.

In this study, 47 compounds were detected and tentatively identified in the infusion of *A. copa* (Figure 2). Previously, from a Argentinean sample of this plant *p*-coumaric acid, luteolin, chrysoeriol, and vitexin were reported, to present vasodilation (Gorzalczany et al., 2013b) also, some flavonoids were isolated from the sample: spinacetin, tricin, jaceosidin, axillarin, penduletin, and chrysoeriol which showed anti-inflammatory activity (Moscatelli et al., 2006), the compound: 3,5-dihydroxy 6,7,3’,4’-tetramethoxyflavone was isolated previously and presented antimicrobial activity (Morales et al., 2003). Several sesquiterpene lactones such as chrysartemin A, were also reported previously from this species (de la Fuente et al., 1986). In this work several compounds are reported for the first time in this species and the determination of those metabolites in this Chilean sample were characterized based on UV absorption and high-resolution mass spectrometry fragmentation patterns for the first time (Table 1). The content of individual compounds (mg/Kg of dry plant) are depicted in Supplementary Table S1 (Supplementary Material) and the chromatograms are depicted in Supplementary Figure S1 (Supplementary Material). Main compound peak **30** was not quantified, due to lack of standard.

**TABLE 1:** High-resolution UHPLC- MS Identification of metabolites from *Artemisia copa* infusion.

Peak number	UV max (nm)	Tentative identification	Formula [M-H]^-^		Retention time (min)	Theoretical mass (*m/z*)	Measured mass *(m/z)*		Accuracy (ppm)	MS^n^ ions (ppm)
1	272–345	Quinic acid	C_7_H_11_O_6_^-^		1.55	191.05501	191.05569		3.56	173.04527 (C_7_H_9_O_5_^−^) (M^−^-H_2_O); 109.02870 (C_7_H_9_O_5_^−^)
2	371	Isoquinic acid	C_7_H_11_O_6_^-^		1.69	191.05501	191.05557		2.92	111.00788 (C_5_H_3_O_3_^−^)
3	351	Citric acid	C_6_H_7_O_7_^-^		2.07	191.01863	191.01929		3.44	111.00790 (C_5_H_3_O_3_^−^)
4	**-**	2-Hydroxyglutaric acid	C_5_H_7_O_5_^-^		2.39	147.02880	147.02917		2.55	133.01477 (C_4_H_5_O_5_^−^)
5	**-**	Lilioside A	C_11_H_19_O_9_^-^		3.08	295.10236	295.10349		3.81	133.04996 (C_5_H_9_O_4_^−^)
6	246	2,3-Butanediol apiosylglucoside	C_15_H_27_O_11_^-^		3.34	383.15479	383.15598		3.09	129.09160 (C_7_H_13_O_2_^−^)
7	231	Artemisinic acid	C_15_H_21_O_2_^-^		4.09	233.15361	233.15446		3.68	161.04453 (C_6_H_9_O_5_^−^)
8	299	2,4,6-Trihydroxyanisole	C_7_H_7_O_4_^-^		4.85	155.03389	155.03441		3.37	129.09145 (C_7_H_13_O_2_^−^)
9	228	Cinnamyl tiglate	C_14_H_15_O_2_^-^		5.77	215.10666	215.10745		3.70	197.09648 (C_14_H_13_O^−^)
10	**-**	Pleoside	C_15_H_19_O_9_^-^		6.12	343.10346	343.10361		3.63	—
11	-	Methyl vanillate glucoside	C_15_H_19_O_9_^-^		7.71	343.10236	343.10352		3.37	—
12	217–281	Rehmaionoside A	C_19_H_33_O_8_^-^		8.64	389.21809	389.21957		3.38	181.05011 (C_9_H_9_O_4_^−^)
13	221–284	Gaultherin	C_19_H_25_O_12_^-^		9.01	445.13405	445.13528		2.76	374.16104, 199.09721
14	222–285	4-Hydroxybenzoic acid	C_7_H_5_O_3_^-^		9.19	137.02442	137.02379		3.41	—
15	—	2-Isopropylmalic acid	C_7_H_11_O_5_^-^		9.48	175.06010	175.06079		3.94	—
16	218–325	Chlorogenic acid	C_16_H_17_O_9_^-^		9.74	353.08781	353.08789		3.34	191.05571 (C_7_H_11_O_6_^−^)
17	223–409	7-Demethylsuberosin	C_14_H_13_O_3_^-^		10.13	229.08592	229.08670		3.40	135.04437 (C_8_H_7_O_2_^−^)
18	270-329-427	Dihydro-*p*-coumaroylglucose	C_15_H_19_O_8_^-^		10.33	327.10744	327.10855		3.47	147.04445 (C_9_H_7_O_2_^−^)
19	259–431	Schaftoside	C_26_H_27_O_14_^-^		10.52	563.13953	563.14020		1.18	374.16104
20	269–346	Kaempferol-3-*O*-glucoside	C_21_H_19_O_11_^-^		10.77	447.09219	447.09329		2.47	175.03952
21	226–330	Feruloyl arabinobiose	C_20_H_25_O_12_^-^		10.88	457.13405	457.13535		2.82	215.10750 (C_14_H_15_O_2_^−^)
22	218–326	3-*O*-Feruloylquinic acid	C_17_H_19_O_9_^-^		11.06	367.10236	367.10364		3.48	173.04486 (C_7_H_9_O_5_^−^)
23	213–338	Apigenin 7-*O*-glucoside	C_21_H_19_O_10_^-^		11.30	431.09727	431.09845		2.74	197.09671 (C_14_H_13_O^−^)
24	225-309-	Kaempferol 7-rhamnoside	C_21_H_19_O_10_^-^		11.44	431.09727	431.09851		2.87	163.03955 (C_9_H_7_O_3_^−^)
25	268–342	Kaempferol-3-*O*-galactoside	C_21_H_19_O_11_^-^		11.52	447.09219	447.09341		2.74	285.04037 (C_15_H_9_O_6_^−^; kaempferol)
26	296–439	Cynarine	C_25_H_23_O_12_^-^		11.81	515.11840	515.11932		1.78	135.04436 (C_8_H_7_O_2_^−^)
27	265–354	Kaempferol-3-*O*-acetyl-glucoside	C_23_H_21_O_12_^-^		12.12	489.10275	489.10376		2.05	285.04028 (C_15_H_9_O_6_^−^; Kaempferol)
28	336–462	Nepochlorogenic acid 1,3 di-*O*-caffeoyl quinic acid	C_25_H_23_O_12_^-^		12.23	515.11840	515.11945		2.03	191.05562 (C_7_H_11_O_6_^−^)
29	231-332-462	Hydroxyoctanoic acid-*O*-Glucoside	C_14_H_25_O_8_^-^		12.33	321.15439	321.15564		3.87	159.10207 (C_8_H_15_O_3_^−^; hydroxyoctanoic acid)
30	250–326	Chrysartemin A	C_15_H_17_O_5_^-^		12.51	277.10705	277.10809		3.75	135.08081 (C_9_H_11_O^−^)
31	236	Unknown	C_13_H_13_O_15_^-^		13.05	409.02599	409.02380		-2.68	181.08667 (C_10_H_13_O_3_^−^)
32	228–427	trans-β-Damascenone	C_13_H_17_O^-^		13.87	189.12739	189.12820		4.29	145.02859 (C_9_H_5_O_2_^−^)
33	232–309	Unknown	C_13_H_15_O_4_^-^		14.14	235.09649	235.09746		4.13	145.02879 (C_9_H_5_O_2_^−^)
34	265–354	Kaempferol	C_15_H_19_O_6_^-^		14.36	285.03936	285.04059		4.29	135.04439 (C_8_H_7_O_2_^−^)
35	239–340	3,7-Dimethylquercetagetin	C_17_H_13_O_8_^-^		15.37	345.06049	345.06183		3.89	129.09175 (C_7_H_13_O_2_^−^)
36	290–396	4-Propionylbenzoic acid	C_10_H_9_O_3_^-^		15.78	177.05462	177.05531		3.91	145.02884 (C_9_H_5_O_2_^−^)
37	239–382	Peuarenarine	C_24_H_25_O_8_^-^		16.68	441.15439	441.15570		2.96	145.02876 (C_9_H_5_O_2_^−^)
38	237–345	7, 3’-dimethoxyquercetin	C_17_H_13_O_7_^-^		17.36	329.06558	329.06683		3.81	299.01971 (C_15_H_7_O_7_^−^; quercetin)
39	233–340	3-*O*-Methylkaempferol	C_16_H_11_O_6_^-^		17.77	299.05501	299.05627		4.21	134.03671 (C_8_H_6_O_2_^−^)
40	255–367	Eupatolitin	C_17_H_13_O_8_^-^		18.28	345.06159	345.06189		4.04	183.08127 (C_13_H_11_O^−^)
41	256–349	Irigenin	C_18_H_15_O_8_^-^		18.90	359.07724	359.07748		3.70	329.02969 (C_16_H_9_O_8_^−^)
42	262–425	Myristicin	C_11_H_11_O_3_^-^		19.01	191.07027	191.07101		3.89	145.02888 (C_9_H_5_O_2_^−^)
43	241–312	Calodendrolide	C_15_H_15_O_4_^-^		19.47	259.09758	259.09753		4.04	145.02895 (C_9_H_5_O_2_^−^)
44	269–338	Eupatorin	C_18_H_15_O_7_^-^		20.14	343.08123	343.08264		4.11	215.10728 (C_14_H_15_O_2_^−^)
45	254–354	3,6,7,8,3'-tetramethoxymyricetin	C_19_H_17_O_8_^-^	20.27		373.09289	373.09320	3.76		129.09129 (C_7_H_13_O_2_^−^)
46	248–392	Pentyl benzoate	C_12_H_15_O_2_^-^		20.95	191.10666	191.10741		3.94	427.21265(C_25_H_31_O_6_^−^)
47	291–396	5,7-Dimethoxy-2,2-dimethylchromene	C_13_H_15_O_3_^-^		21.30	219.10157	219.10248		4.14	145.02876 (C_9_H_5_O_2_^−^)

#### Sugars and Hydrocarbons

Peak **1** with a pseudo-molecular ion at *m/z*: 191.05569 was identified as quinic acid (C_7_H_11_O_6_^−^) and its isomer peak **2** as isoquinic acid at *m/z* 191.05557. Peak **3** was identified as citric acid (C_6_H_7_O_7_^−^). Peak **4** with a molecular ion at *m/z*: 47.02917 was identified as 2-hydroxyglutaric acid (C_5_H_7_O_5_^−^). peak **12** as rehmaionoside A (C_19_H_33_O_8_^−^), a constituent of *Rehmannia glutinosa* (Gaertn.) DC. (Liu et al., 2020).

#### Phenolic Acids

Peak **5** with a [M-H]^−^ ion at *m/z*: 295.10349 was identified as the coumaroyl glycerol lilioside A (C_11_H_19_O_9_^−^) (Kaneda, 1990), peak **6** was identified as 2,3-butanediol apiosylglucoside (383.15598, C_15_H_27_O_11_^−^). Peak **8** with a [M-H]^−^ ion at *m/z* 155.03441 was identified as 2,4,6-trihydroxyanisole (C_7_H_7_O_4_^−^). Peak **9** was identified as cinnamyl tiglate (C_14_H_15_O_2_^−^), peak **10** with a molecular ion at *m/z*: 343.10361, was identified as pleoside (C_15_H_19_O_9_^−^) this compound was reported antiproliferative together with chlorogenic acid (Jafari et al., 2017), peak **11** as its isomer methyl vanillate glucoside, Peak **13** as gaultherin (C_19_H_25_O_12_^−^). Peak **14** with a [M-H]^−^ ion at *m/z*: 137.02379 was identified as 4-hydroxybenzoic acid (C_7_H_5_O_3_^−^), peak **15** as 2-isopropylmalic acid (C_7_H_11_O_5_^−^). Peak **19** as schaftoside (C_26_H_27_O_14_^−^) peak **16** and **28** were identified as isomers of chlorogenic acid (C_16_H_17_O_9_^−^), peak **17** as 7-demethylsuberosin (C_14_H_13_O_3_^−^), and peak **18** as dihydro-*p*-coumaroylglucose (C_15_H_19_O_8_^−^), respectively. Peak **21** as feruloyl arabinobiose (457.13535), Peak **22** with a [M-H]^−^ ion at *m/z*: 367.10364 was identified as 3-*O*-feruloylquinic acid (C_17_H_19_O_9_^−^), peak **26** as cynarine, peak **32** as trans-β-damascenone (C_13_H_17_O^−^), and peak **36** as 4-propionylbenzoic acid (177.05531) and **37** as peuarenarine (C_24_H_25_O_8_^−^), peak **46** as pentyl benzoate (C_12_H_15_O_2_^−^) and finally, peak **47** as 5,7-dimethoxy-2,2-dimethylchromene (C_13_H_15_O_3_^−^).

#### Fatty Acids

Peak **29** was identified as hydroxyoctanoic acid-*O*-glucoside (C_14_H_25_O_8_^−^)

#### Flavonoids

Peak **20** with a [M-H]^−^ ion at *m/z*: 447.09372 was identified as kaempferol-3-*O*-glucoside (C_21_H_19_O_11_^−^) while peak **23** with a pseudo-molecular ion at *m/z* 431.09845 was identified as apigenin 7-*O*-glucoside (C_21_H_19_O_10_^−^) (Taşkın et al., 2018) and peak **24** as kaempferol 7-rhamnoside (C_21_H_19_O_10_^−^). Peak **25** as kaempferol-3-*O*-galactoside (C_21_H_19_O_11_^−^) and peak **27** with a molecular ion at *m/z*: 489.10376 as kaempferol-3-*O*-acetyl-glucoside (C_23_H_21_O_12_^−^) a constituent of *Moringa oleifera* Lam. leaves (Mousa et al., 2019), finally, peaks **34**, **35** and **38**–**42** and **44**, **45** as: kaempferol (C_15_H_19_O_6_^−^), 3,7-dimethylquercetagetin (C_17_H_13_O_8_^−^), 7,3’-dimethoxyquercetin (C_17_H_13_O_7_^−^), 3-*O*-methylkaempferol (C_16_H_11_O_6_^−^), eupatolitin (C_17_H_13_O_8_^−^), irigenin (C_18_H_15_O_8_^−^), myristicin (C_11_H_11_O_3_^−^), eupatorin (C_18_H_15_O_7_^−^), and 3,6,7,8,3'-tetramethoxymyricetin (C_19_H_17_O_8_^−^), respectively.

#### Sesquiterpene Lactones

Artemisinic acid peak **7** (C_15_H_21_O_2_^−^), main compound in famous Chinese medicinal plant *Artemisia annua L.* (Bora and Sharma, 2011), was found and identified with a molecular ion at *m/z*: 233.15446, and peak **30** as chrysartemin A (C_15_H_17_O_5_^−^) also constituent of the *Artemisia* genus.

### Total Phenolic Content, Total Flavonoid Content, and Antioxidant Activity

*Artemisia copa* infusions were assessed *in vitro* for total phenolic content (TPC) and total flavonoid content (TFC) also, DPPH, FRAP, and ORAC assays were used to determine the antioxidant properties and are summarized in Table 2. These *in vitro* assays are simple and widely employed for the evaluation of antioxidant power and ROS quenching (Moon and Shibamoto, 2009). These results were compared to another very used Andean altiplano plant, *Aloysia deserticola* (Phil.) Lu-Irving and O'Leary (syn. *Acantholippia deserticola* Phil.) (Verbenaceae) previously reported by us (Larrazabal et al., 2018)*.* Phenolic compounds are structurally diverse and comprise phenolic acids, flavonoids, tannins, coumarins, chalcones, iridoids, among others, most of them bearing phenolic moieties that give them antioxidant and beneficial health properties. In this study three main classes of compounds were tentatively identified: organic acids, feruloyl and coumaroyl derivatives, plus flavonoids. In addition, 47 compounds were detected in their corresponding infusion, that could be responsible for the bioactivities reported. The results observed for TPC and TFC of *A. copa* infusion were 155.6 ± 2.9 mg/g and the 5.5 ± 0.2 mg/g of dry plant, respectively. On the other hand, DPPH was performed to know the bleaching capacities of the infusion, and showed an IC_50_ of 89.72 ± 0.01 µg/ml infusion. While the ABTS and DPPH values for *A. deserticola* infusions were 337 and 438 µg/ml (Larrazabal et al., 2018). Also, the FRAP assay was conducted giving the lower antioxidant power obtained (6.2 ± 0.2 mg trolox equivalent per 100 g of the dry plant.) compared to the famous infusions of the arid plant *A. deserticola* (Larrazabal et al., 2018). This assay is based on the ability to reduce Fe^+3^ to Fe^+2^ by antioxidants in a sample. Regarding the ORAC assay, the infusion value was 1989 ± 5.2 μM Trolox equivalents (TEAC) per 100 g of the dry plant. These values can classify these *A. copa* infusions as moderate antioxidants properties, like edible fruits such as cherries, strawberries, and plums (Rodríguez et al., 2014; Ramirez et al., 2015).

**TABLE 2:** Total phenolics, total flavonoids, and antioxidants activity of *Artemisia copa* infusion.

Assay	*Artemisia copa* infusion	Standard
Total phenolics^a^	155.6 ± 2.9	—
Total flavonoids^b^	5.5 ± 0.2	—
FRAP^c^	6.2 ± 0.2	—
ORAC^d^	1989 ± 5.2	—
DPPH^e^	89.72 ± 0.01	Gallic acid: 0.55 ± 0.01

All values were expressed as means ± SEM (*n* = 3).

FRAP, ferric reducing/antioxidant power; ORAC, Oxygen Radical Absorbance Capacity; DPPH, 2,2-diphenyl-1-picryl-hydrazyl-hydrate. All values in the column are significantly different (at *p* < 0.05).

a expressed in mg gallic acid equivalent per g of dry plant.

b expressed in mg quercetin per g of dry plant.

c expressed in mg trolox equivalent per 100 g of the dry plant.

d expressed in μM Trolox equivalents per 100 g of the dry plant.

e expressed as IC_50_ in µg of extract or standard per ml.

### Cholinesterase Inhibitory Activity

In this study, *Artemisia copa* infusion was assessed for *in vitro* inhibitory activity against AChE and BChE using galantamine as the positive control (reference: galantamine 0.26 ± 0.03 μg/ml). The results are summarized in Table 3 and are expressed as IC_50_ values (µg/ml). The inhibition of key enzymes has been a very useful strategy to know the pharmacological potential of herbal medicines. Moreover, inhibition of enzymes linked to Alzheimer's disease (AD), such AChE and BChE (Pan et al., 2013). In the AChE assays*, A. copa* showed an IC_50_ of 3.92 ± 0.08 µg/ml, while in the BChE assays the IC_50_ results were 44.13 ± 0.10 µg/ml respectively. In previous reports the cholinesterase inhibitory activity of Algerian *Artemisia* spp: *A. absinthium* L.*, A. fragrans* Willd. and *A. herba-alba* Asso were evaluated and showed similar results against AChE but not activity against BChE (Orhan et al., 2010), also Turkish *Artemisia, A. absinthium* was reported (Zengin et al., 2017). *A. copa* possessed the capacity to inhibit AChE and BChE enzymes.

**TABLE 3:** Cholinesterase inhibitory activity of *Artemisia copa* infusion.

Assay	*Artemisia copa* infusion (IC_50_)	Galantamine (IC_50_)
AChE	3.92 ± 0.08	0.26 ± 0.03
BChE	44.13 ± 0.10	3.82 ± 0.02

All values were expressed as means ± SEM (*n* = 3). IC_50_ as expressed in µg per ml. AChE, Acetylcholinesterase; BChE, Butyrylcholinesterase. Values in the same column marked with the same letter are not significantly different (at *p* < 0.05).

Regarding the constituents identified in the infusion of *A. copa*, the literature has indicated that, for example, the increase of brain AChE activity was reported for citric acid, acting as a neuroprotective agent in animal models (Abdel-Salam et al., 2016). Regarding hydroxybenzoic acid, have evaluated the antioxidants and ChE inhibitors properties of hydroxybenzoic acid derivatives as dual-targets (Oliveira et al., 2018). In a previous report phenolic acids from malt, included hydroxybenzoic acid, showed efficient properties against AChE and BChE respectively (Szwajgier and Borowiec, 2012). Chlorogenic acid exert protective effects on learning and memory impairment. Furthermore, *ex vivo* experiments showed inhibition of AChE activity in hippocampus and frontal cortex. On the other hand, *in vitro* experiments inhibit AChE activity with a IC_50_ = 98.17 µg/ml (Kwon et al., 2010). Also, the combination of chlorogenic acid with caffeic acid displayed an antagonist response against AChE and BChE. The neuroprotective properties have been associated to the ChE inhibition, as well to the prevention of oxidative damage (Oboh et al., 2013). AChE activity have been reported for **7-**demethylsuberosin isolated from genus *Angelica*, suggesting a potential agent for the AD (Kang et al., 2001; Kwon et al., 2017). In addition, Schaftoside isolated from the water layer of *Lycopodiella cernua* (L.) Pic.Serm. showed strong inhibitory activity against BChE (Hung et al., 2015).

The *in vitro* ChE inhibitory activities of different flavonoids derivatives (rutin, quercetin and kaempferol-3-*O*-galactoside) have been previously investigated. Docking experiments of quercetin showed a competitive inhibition mechanism against both enzymes (Khan et al., 2009). The principal active compound from *Cynara scolymus* L., cynarine, was purified from *Onopordum illyricum* L. and showed antioxidant, antiradical and inhibitory activities against AChE (Topal et al., 2016). On the other hand, the isoflavonoid irigenin was tested against AChE and BChE. The results showed a moderate activity against BChE (less to 40% of inhibition) (Ślusarczyk et al., 2019). Besides, inhibitory activity of AChE of myristicin have been investigated *in vitro* and *in vivo* experiments. The enzymatic results indicate that myristicin caused competitive and noncompetitive inhibition (Jaiswal et al., 2010). Finally, the liminoid calodrendolide showed neuroprotective effects against glutamate-induced neurotoxicity in rat cortical cells (Jeong et al., 2008).

This result suggests that infusion from *A. copa* could be explored as an alternative medicine for AD.

### Molecular Docking Studies

To get insights on the intermolecular interactions, four flavonoids contained in the *Artemisia copa* infusion: apigenin-7-*O*-glucoside, kaempferol-3-*O*-galactoside, kaempferol-3-*O*-acetyl-glucoside and kaempferol-7-rhamnoside (which are in high amount and proportion in this species), as well as the known cholinesterase inhibitor galantamine (Supplementary Figure S2), were selected and subjected to molecular docking studies over AChE (*Tc*AChE) and BChE (*h*BChE) (Full description, Supplementary Material)*.* The relationship between flavonoids and their potential as cholinesterase inhibitors have been reported (Sevindik et al., 2015; Khan et al., 2018). Table 4 shows the binding energies expressed in kcal/mol of every compound mentioned above.

**TABLE 4:** Binding energies obtained from docking experiments of selected flavonoids and Galantamine over cholinesterases *Tc*AChE and *h*BChE.

Compounds	Binding energy (kcal/mol) Acetylcholinesterase (*Tc*AChE)	Binding energy (kcal/mol) Butyrylcholinesterase (*h*BChE)
Apigenin-7-*O*-glucoside	9.76	−5.93
Kaempferol-3-*O*-galactoside	−2.04	−8.92
Kaempferol-3-*O*-acetyl-glucoside	−1.27	−8.36
Kaempferol-7-rhamnoside	12.66	−3.38
Galantamine	−11.81	−9.50

**FIGURE 3:**
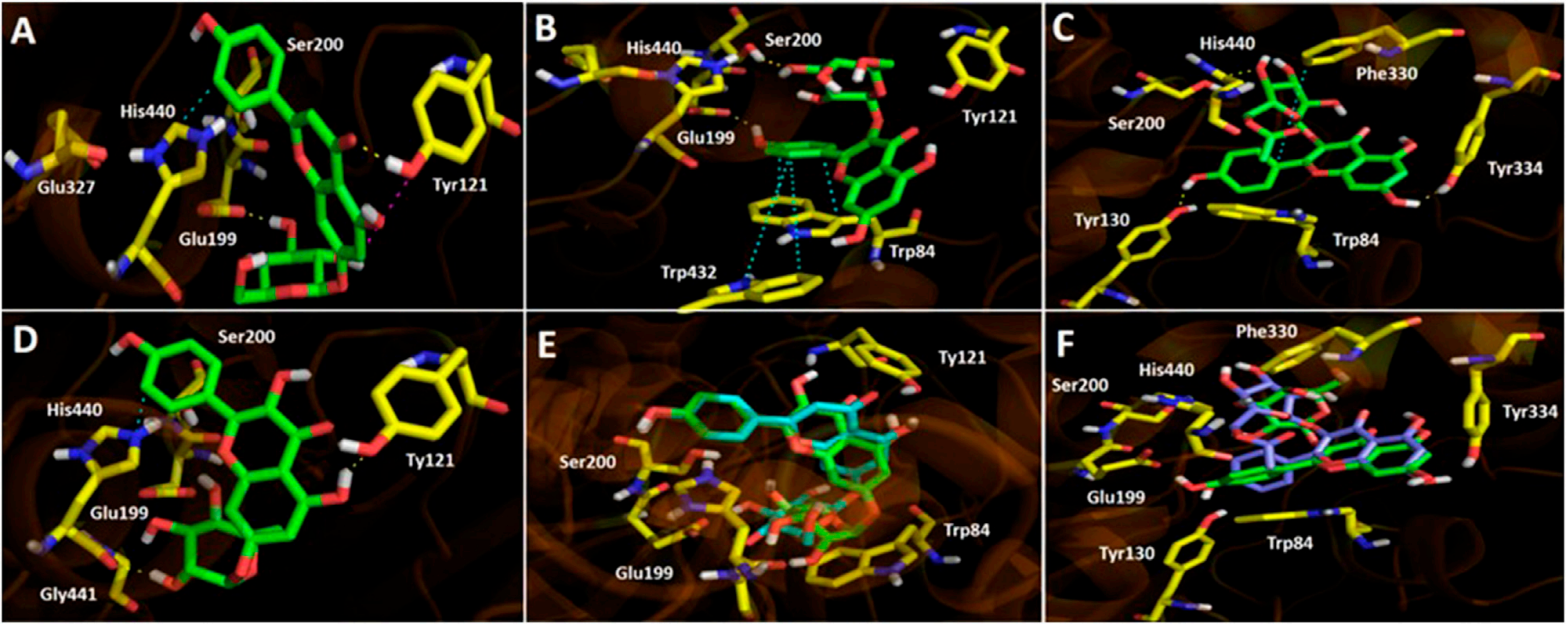
Predicted binding mode and predicted intermolecular interactions among selected flavonoids and the residues of *Torpedo californica* acetylcholinesterase (*Tc*AChE) catalytic site. **(A)** Apigenin 7-*O*-glucoside into the catalytic site (two H-bondings with Glu199 and Tyr121; T-Shaped with Tyr121 and π-π with His440). **(B)** Kaempferol-3-*O*-galactoside into the catalytic site (two H-bondings with Glu199 and Ser200; two π-π interactions with Trp84 and Trp432). **(C)** Kaempferol-3-*O*-acetyl-glucoside into the catalytic site (three H-bondings with Tyr130, Tyr334 and His440; one π-π interaction with His440). **(D)** Kaempferol-7-rhamnoside into the catalytic site (two H-bondings with Tyr121 and Gly441; one π-π interaction with His440). **(E)** Apigenin 7-*O*-glucoside (green) and kaempferol-7-rhamnoside (cyan) overlapped into the catalytic site. **(F)** Kaempferol-3-*O*-galactoside (green) and kaempferol-3-*O*-acetyl-glucoside (magenta) overlapped into the catalytic site.

In *Tc*AChE the binding energy descriptors for flavonoids kaempferol-3-*O*-galactoside and kaempferol-3-*O*-acetyl-glucoside turned out to be better than for flavonoids apigenin-7-*O*-glucoside and kaempferol-7-rhamnoside. Apigenin-7-*O*-glucoside, as well as kaempferol-7-rhamnoside displayed deficient binding energies of 9.76 kcal/mol and 12.66 kcal/mol respectively, compared to -2.04 kcal/mol of kaempferol-3-*O*-galactoside and −1.27 kcal/mol of kaempferol-3-*O*-acetyl-glucoside, suggesting that the inhibitory activity over the enzyme lies predominantly in the first two flavonoids mentioned above. This could be explained due flavonoids kaempferol-3-*O*-galactoside and kaempferol-3-*O*-acetyl-glucoside are arranged in similar manners into the catalytic site of the enzyme. In the same way, apigenin-7-*O*-glucoside and kaempferol-7-rhamnoside are also closely overlapped between them. Therefore, both pairs of compounds carry out different interactions with the amino acids, although derivatives kaempferol-3-*O*-galactoside and apigenin-7-*O*-glucoside performed hydrogen bond interactions with the amino acid Glu199 (Figure 3). The differences in the abilities to inhibit the enzyme and the differences in the binding energies of the four flavonoids, must be due because of the greatest amount of hydrogen bond interactions performed by kaempferol-3-*O*-galactoside and kaempferol-3-*O*-acetyl-glucoside, as well as the π-π interactions (especially compared to T-shaped interaction in apigenin-7-*O*-glucoside and only one π-π interaction in kaempferol-7-rhamnoside) performed by the phenyl ring of the 4*H*-chromen-4-one in kaempferol-3-*O*-galactoside with the indole rings of Trp84 and Trp432 and with the same phenyl moiety in kaempferol-3-*O*-acetyl-glucoside and Phe330 of the *Tc*AChE. The hydrogen bonding and the π-π interactions mentioned above must be stronger in kaempferol-3-*O*-galactoside and kaempferol-3-*O*-acetyl-glucoside than those for apigenin-7-*O*-glucoside and kaempferol-7-rhamnoside. For example, Trp bears electron-deficient aromatic rings, whereas the phenyl ring of kaempferol-3-*O*-galactoside possesses an electron-donating hydroxyl group (−OH) causing a stronger interaction than apigenin-7-*O*-glucoside.

**FIGURE 4:**
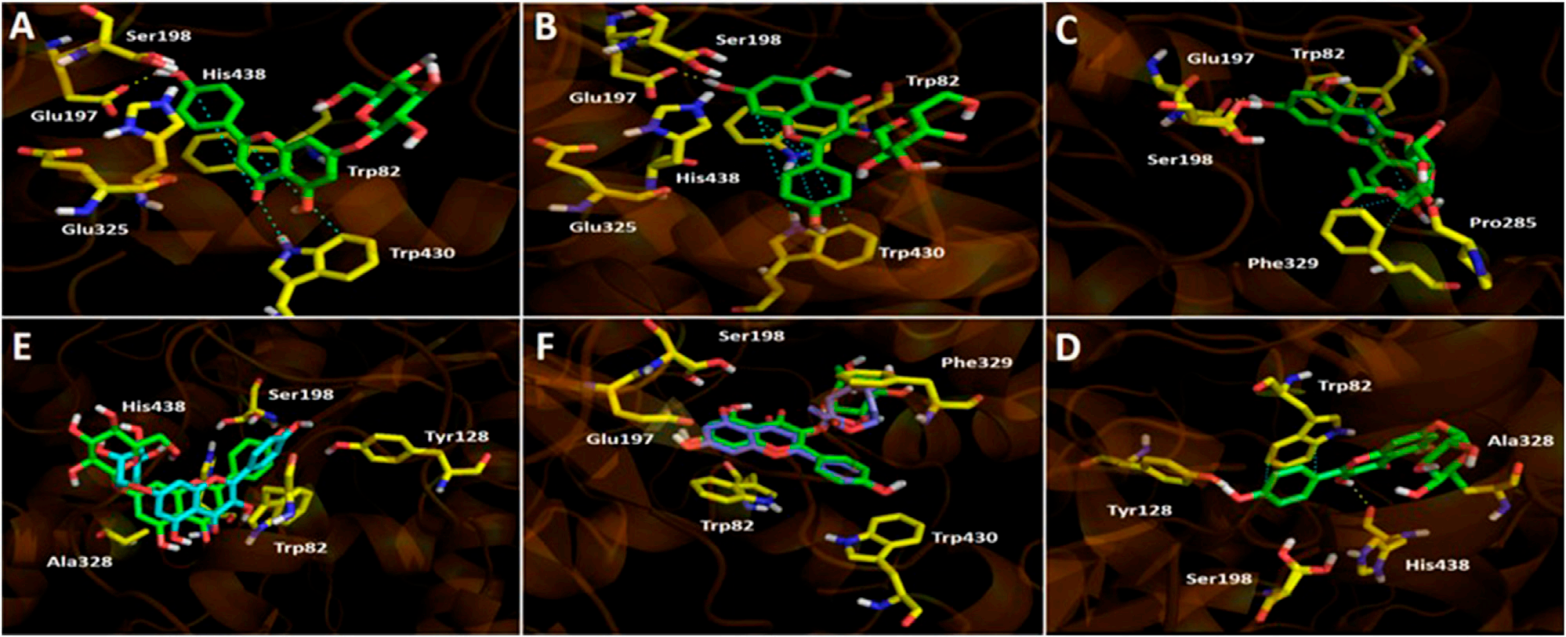
Predicted binding mode and predicted intermolecular interactions among apigenin 7-*O*-glucoside, kaempferol-3-*O*-galactoside, kaempferol-3-*O*-acetyl-glucoside, and kaempferol-7-rhamnoside and the residues of human butyrylcholinesterase (*h*BuChE) catalytic site. **(A)** Apigenin 7-*O-*glucoside into the catalytic site (one H-bond interaction with Glu197; one π-π with Trp430). **(B)** Kaempferol-3-*O*-galactoside into the catalytic site (one H-bond interaction with Glu197; two π-π interactions with Trp82 and Trp430). Kaempferol-3-*O*-acetyl-glucoside into the catalytic site (two H-bondings with Glu197 and Pro285; three π-π interaction with Trp82 and Phe329). **(C)** Kaempferol-3-O-acetyl-glucoside into the catalytic site (two H-bondings with Glu197 and Pro285; three π-π interaction with Trp82 and Phe329). **(D)** Kaempferol-7-rhamnoside into the catalytic site (two H-bondings with Tyr128 and His438; one π-π interaction with Trp82). **(E)** Apigenin 7-*O*-glucoside (green) and kaempferol-7-rhamnoside (cyan) overlapped into the catalytic site. **(F)** Kaempferol-3-*O*-galactoside (green) and kaempferol-3-*O*-acetyl-glucoside (magenta) overlapped into the catalytic site.

Docking results on *h*BChE for the four flavonoids described above, behaved similarly than in *Tc*AChE. The binding energies of kaempferol-3-*O*-galactoside and kaempferol-3-*O*-acetyl-glucoside showed to be better (−8.92 and −8.36 kcal/mol respectively) compared to apigenin-7-*O*-glucoside and kaempferol-7-rhamnoside (−5.93 and −3.38 kcal/mol), suggesting once again that the inhibitory activity over *h*BChE lies predominantly in those derivatives which bear the sugar moieties at position 3- of the 4*H*-chromen-4-one core. The binding modes of compounds into the catalytic site of *h*BChE were different this time related to results seen in *Tc*AChE. Kaempferol-3-*O*-galactoside and kaempferol-3-*O*-acetyl-glucoside are completely overlapped, as well as apigenin-7-*O*-glucoside and kaempferol-7-rhamnoside are arranged in the same manner with a slightly difference. This feature makes that the 4*H*-chromen-4-one moieties of the first two flavonoids to be in opposite positions related to the second ones (Figure 4). Binding energies for *h*BChE turned out to be better than for *Tc*AChE in docking experiments, what is partially divergent with our experimental inhibition assays, where the *A. copa* infusion showed an IC_50_ = 3.92 ± 0.09 µg/ml for *Tc*AChE and an IC_50_ = 44.13 ± 0.10 µg/ml for *h*BChE.

### Antiprotozoal Activity Evaluation.

To assess the potential activity from *Artemisia copa* infusion, we have performed a screening test against *Trypanosoma cruzi* and *Leishmania amazonensis*. Protozoan parasites are a significant cause of infectious diseases with high morbidity and mortality. Besides, Chagas and Leishmaniasis diseases have a great impact on developing countries, and no new treatments have been developed in the last 40 years (Costa et al., 2018). *Artemisia copa* showed trypanocidal activity against *Trypanosoma cruzi* with a LD_50_ of 131.8 µg/ml and against *Leishmania amazonensis* with a LD_50_ of 457 µg/ml (Supplementary Table S2, Supplementary Figure S3, Supplementary Material). Artemisinic acid is a sesquiterpene isolated from *A. annua* and a very important precursor for the production of artemisinin (Kong et al., 2013). Moreover, the antiprotozoal properties of artemisinin derivatives have been reviewed in detail (Loo et al., 2017). Asteraceae family plants play an important contribution to the development of new trypanocidal and leishmanicidal agents (Moraes Neto et al., 2019). The *Artemisia* genus has been pointed out as interesting antiprotozoal agents (Emami et al., 2012). The essential oils from *Artemisia campestris* L. and *Artemisia herba-alba* showed IC_50_ of 44 and 68 µg/ml against *Leishmania infantum* (Aloui et al., 2016). While, *Artemisia annua* showed IC_50_ of 22 µg/ml against *Leishmania donovani* (Sen et al., 2007; van der Kooy and Sullivan, 2013). The results of *in vitro* studies indicate that the infusion from *Artemisia copa* displayed low trypanocidal activity against *Trypanosoma cruzi* and very low activity against *Leishmania amazonensis*. The antiprotozoal properties of the flavonoid eupatorin isolated from the genus *Stevia* (Asteraceae) was investigated. Eupatorin displayed activity against *Tripanozoma cruzi* epimastigotes and trypomastigotes. Additionally, the activity against *Leishmania braziliensis* and the cytotoxicity on Vero cells were evaluated (Beer et al., 2016). The antiprotozoal properties of 7**-**demethylsuberosin isolated from the bark of *Citropsis articulata* (Willd. ex Spreng.) Swingle and M.Kellerm. (Rutaceae) was reported against *Plasmodium falciparum* (Lacroix et al., 2011). The antiprotozoal properties of the infusion of *A. copa* has not been previously reported and additional studies are necessary to explore the antiprotozoal activities.

## Conclusion

The potential antioxidant, antiprotozoal, and cholinesterase inhibition activity plus the chemical profiling of *Artemisia copa* infusion were assessed. UHPLC-MS were used for the first time to detected 47 compounds in the infusion of the Altiplano plant, and to the best of our knowledge, is the first investigation on the cholinesterase inhibition activity. Our docking studies suggest that the inhibitory activity over the enzymes lies predominantly on kaempferol‐3‐O‐galactoside and kaempferol‐3‐O‐acetyl‐glucoside. *A. copa* could be recommended as a supplementary beverage with cholinesterase inhibition potentials. The antiprotozoal evaluation showed no significant results against *T. cruzi* and *L. amazonensis*. However, should be considered that activity was evaluated in extracts where the potential active product(s) against these protozoa would be highly diluted. This study may contribute to a better understanding of the chemistry and biological activity of this plant.

## Data Availability Statement

The datasets presented in this study can be found in online repositories. The names of the repository/repositories and accession number(s) can be found in the article/Supplementary Material.

## Author Contributions

MS, JG, MJL-F, JE, and C F-G wrote and revised the manuscript, JR-P, MJL-F, AG, and AP analyzed the data, AP, and JP-R performed the antioxidant assays, JG and KS performed the antiprotozoal assays.

## Funding

This research received funds from FONDECYT regular 1180059, ML-F received funds from MINEDUC-UA project code ANT 1856. CF-G acknowledges post doctorate grant 3190794. JE gratefully acknowledges funding from CONICYT (PAI/ACADEMIA 79160109

## Conflict of Interest

The authors declare that the research was conducted in the absence of any commercial or financial relationships that could be construed as a potential conflict of interest.

## Abbreviations

AAPH, 2,2'-azobis(2-amidinopropane) dihydrochloride; AChE, acetylcholinesterase; ANOVA, one-way analysis of variance; ATCI, acetyl-thiocholine iodide; BChE, butyrylcholinesterase; ChE, cholinesterase; DPPH, 2,2,1-diphenyl-1-picrylhydrazyl; DTNB, 5-dithio-bis(2-nitrobenzoic) acid; FRAP, ferric reducing antioxidant power; GAE, gallic acid equivalent; IC50, half maximal inhibitory concentration; MS, mass spectrometry; NO, nitric oxide; ORAC, oxygen radical absorbance capacity; OT, orbitrap technology; PDA, photodiode array detection; PTFE, polytetrafluoroethylene; QE, quercetin equivalent; ROS, reactive oxygen species; RP, reverse phase; TE, trolox equivalents; TEAC, trolox equivalent antioxidant capacity; TFC, total flavonoid content; TIC, total ion chromatogram; TPC, total phenolic content; TPTZ, 2,4,6-tri(2-pyridyl)-s-triazine; Trolox, 6-hydroxy-2,5,7,8-tetramethylchromane-2-carboxylic acid; UV, ultraviolet; UHPLC, ultra high performance chromatography; HESI II, electrospray heated ionization probe.
