# Supplementary material for: Chemical Profiling, Antioxidant, Anticholinesterase, and Antiprotozoal Potentials of Artemisia copa Phil. (Asteraceae)
Source: Front Pharmacol. 2020 Dec 4;11:594174. doi: 10.3389/fphar.2020.594174 (PMC7746865; doi:10.3389/fphar.2020.594174)
Supplement: Supplementary file 1 [file datasheet1.docx]

**SUPPLEMENTARY MATERIAL**

**Chemical profiling, antioxidant, anticholinesterase, and antiprotozoal potentials of *Artemisia copa* Phil. *(*Asteraceae)**

**María José Larrazábal-Fuentes^1†^, Carlos Fernández-Galleguillos^2†^, Jenifer Palma-Ramírez^1^, Javier Romero-Parra^3^, Kevin Sepúlveda^4^,** **Alexandra Galetovic^5^, Jorge González^4^, Adrián Paredes^6^, Jorge Bórquez^6^, Mario J. Simirgiotis^2*^, Javier Echeverria^7^***

^1^Unidad Alimentos, Departamento de Ciencias de los Alimentos y Nutrición, Facultad de Ciencias de la Salud, Universidad de Antofagasta, Antofagasta, Chile

^2^Instituto de Farmacia, Facultad de Ciencias, Universidad Austral de Chile, Valdivia, Chile.

^3^Departamento de Química Orgánica y Fisicoquímica, Facultad de Ciencias Químicas y Farmacéuticas, Universidad de Chile, Santiago, Chile

^4^Unidad de Parasitología Molecular, Departamento de Tecnología Médica, Facultad de Ciencias de la Salud, Universidad de Antofagasta, Antofagasta, Chile

^5^Departamento Biomédico, Universidad de Antofagasta, Antofagasta, Chile

^6^Laboratorio de Productos Naturales, Departamento de Química, Facultad de Ciencias Básicas, Universidad de Antofagasta, Antofagasta, Chile.

^7^Departamento de Ciencias del Ambiente, Facultad de Química y Biología, Universidad de Santiago de Chile, Santiago, Chile

*** Correspondence:**Mario J. Simirgiotis. E-mail: mario.simirgiotis@uach.cl.

Javier Echeverría. E-mail: javier.echeverriam@usach.cl

**^†^** These authors share first authorship

1. **HPLC-PDA quantitation of main phenolic compounds in *A. copa.***

Some phenolic compounds were quantified using the UHPLC Dionex 3000 RS with photodiode array detector at flavonoid (255 and 265 nm) and phenolic acid (330 nm) wavelenght as reported previously carried out according to ICH Guideline Q2 (R1) (Barrientos et al., 2020). Some curves for representative compounds, each one covering 6 points from 0.01 to 0.5 mg/mL solutions, in triplicate, and injecting 10 mL. The linearity of the chromatographic method developed was confirmed by the coefficients of determination all around 0.999. For kaempferol based compounds the calibration curve was performed with kaempferol standard, (R^2^= 0.9997) and for quercetin based compounds, the curve was performed with quercetin standard (R^2^= 0.9989), and other phenolic acid were quantified using a chlorogenic acid curve (R^2^= 0.9994). Using PDA, the lowest detection (LOD) and quantification (LOQ) limits, 0.0115 mg/L and 0.0322 mg/L respectively, were obtained for kaempferol, while the highest limits, 0.5543 mg/L, and 1.476 mg/L were for chlorogenic acid. For the quantification the prepared infusion was injected (10 mL) in triplicate at 25 ^o^C. The content of individual compounds (mg/Kg of dry plant) are depicted in **Table S1** and the chromatograms are depicted in **Figure S1**. Main compound peak **30** was not quantified, due to lack of standard.

**Table S1**. Quantitative HPLC-PDA analysis of phenolics compounds in *A. copa*

| **Peak** | **Compound** | **Uv maximum absorbance** | **Quantitation mg/kg** |
| --- | --- | --- | --- |
| **16** | Chlorogenic acid | 235-330 | 75 ± 4 |
| **18** | Dihydro-*p*-coumaroylglucose | 330 | 123 ± 3 |
| **19** | Schaftoside | 255-349 | 53 ± 2 |
| **20** | Kaempferol 7-rhamnoside | 255-349 | 67 ± 3 |
| **22** | Feruloyl-3-*O*-quinic acid | 330 | 28 ± 1 |
| **23** | Apigenin 7-*O*-glucoside | 255-349 | 4322 ± 5 |
| **24** | Kaempferol 7-rhamnoside | 265-365 | 2321 ± 3 |
| **27** | Kaempferol-3-*O*-acetyl-glucoside | 265-365 | 943 ± 3 |
| **28** | Neo-chlorogenic acid | 235-330 | 125 ± 3 |
| **34** | Kaempferol | 265-365 | 76 ± 2 |
| **38** | 7,3’-dimethoxyquercetin | 255-355 | 55 ± 1 |
| **39** | 3-*O*-methylkaempferol | 265-365 | 47 ± 1 |
| **40** | Eupatolitin | 255-355 | 39 ± 2 |
| **42** | Myricitin | 255-355 | 472 ± 5 |
| **44** | Eupatorin | 255-355 | 6 ± 1 |
| **45** | 3,6,7,8,3'-tetramethoxymyricetin | 255-355 | 533±5 |


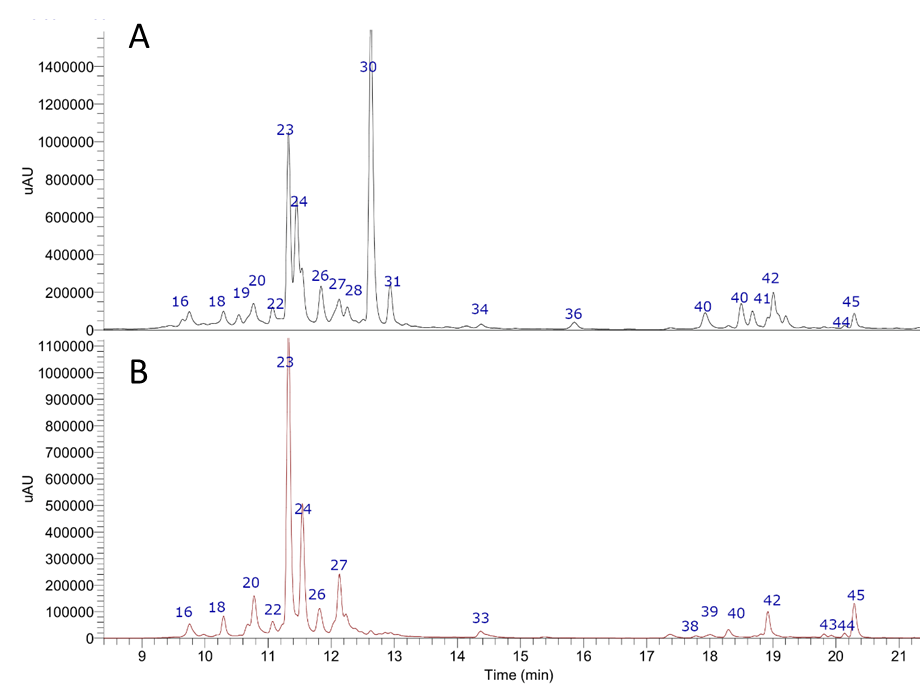


**Figure S1**. PDA Chomatograms of main phenolic compounds. **A**) 255 nm, and **B**) 330 nm.

1. **Docking study**

The geometries and partial charges of major flavonoids apigenin-7-*O*-glucoside, kaempferol-3-*O*-galactoside, kaempferol-3-*O*-acetyl-glucoside, and kaempferol-7-rhamnoside, as well as the known cholinesterase inhibitor Galantamine (**Figure S2**) were fully optimised using the DFT method with the standard basis set PBE0/ 6-311+g*(Adamo and Barone, 1999)(Petersson et al., 1988). All calculations were performed in Gaussian 09W software(Frisch, 2009). Molecular docking simulations over *Torpedo californica* acetylcholinesterase (*Tc*AChE; PDBID: 1DX6 code(Greenblatt et al., 1999)) and human butyrylcholinesterase (*h*BuChE; (Nachon et al., 2013) PDBID: 4BDS code were performed using Autodock 4.2(Morris et al., 2009). Polar hydrogen atoms of both enzymes were added and treated as rigid bodies. Grid maps were calculated using the autogrid option and were centred on the putative catalytic site of each enzyme considering their known residues: Ser200, Glu327 His440 for *Tc*AChE (Sussman et al., 1991) and Ser198, Glu325, His438 for *h*BuChE(Tallini et al., 2018)(Re et al., 1999), respectively. Ser200 of *Tc*AChE catalytic site and Ser198 of *h*BuChE catalytic site were designated as the centre of the grids in each enzyme. The volumes chosen for the grid maps were made up of 60 × 60 × 60 points, with a grid-point spacing of 0.375 Å, which are sufﬁciently large. Docked compound complexes were built using the Lamarckian Genetic Algorithm (Thomsen and Christensen, 2006) which involved 100 runs. The lowest docked-energy binding cluster positions were chosen to be analyzed according to the potential intermolecular interactions between inhibitors and the enzymes. The different complexes were visualised in a Visual Molecular Dynamics program (VMD).


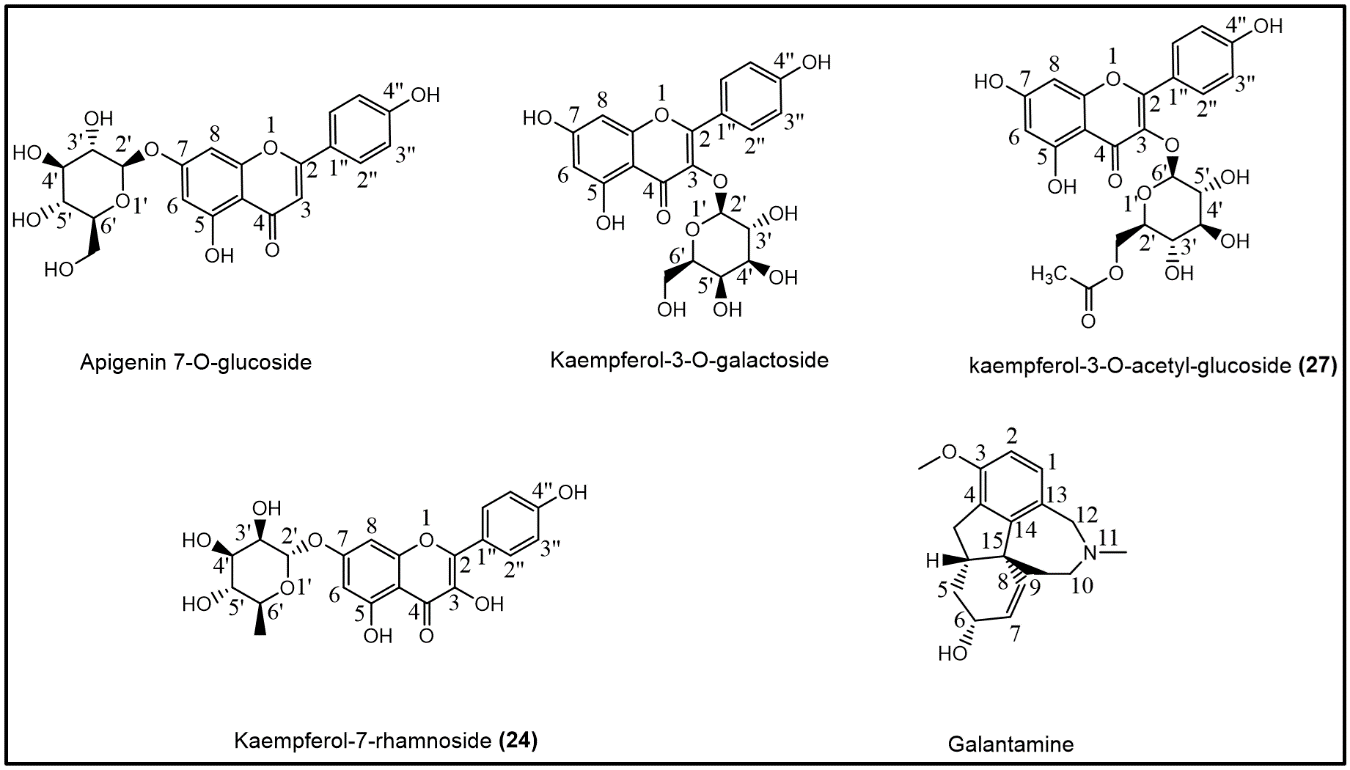


**Figure S2.** Major flavonoids of *A. copa* subjected to docking assays

From 47 compounds, four flavonoids contained in the *Artemisia copa* infusion: apigenin-7-*O*-glucoside, kaempferol-3-*O*-galactoside, kaempferol-3-*O*-acetyl-glucoside, and kaempferol-7-rhamnoside, which are in high amount and proportion in this species, as well as the known cholinesterase inhibitor galantamine (which behaved as the best inhibitor), were subjected to docking assays over *Torpedo californica* acetylcholinesterase (*Tc*AChE; 1DX6) and human butyrylcholinesterase (*h*BuChE; 4BDS). **Table 3** shows the binding energies expressed in kcal/mol of every compound mentioned above.

*Torpedo* electric organ, a major form of AChE, correspond to a homodimer bound to the plasma membrane through covalently attached phosphatidylinositol (Silman and Futerman, 1987). The monomer is an α/β protein with an ellipsoidal shape (Levitt and Chothia, 1976). The enzyme monomer contains 537 amino acids and consist in 12-stranded mixed β-sheet surrounded by 14 α-helices bearing resemblances to several hydrolase structures. The catalytic site of *Tc*AChE consists in a narrow gorge, about 20 Å long, and reaches halfaway into the protein and widens out close to its bottom. The residues involved in the catalytic activity, known as the catalytic triad, are Ser200, Glu327 and His440. Furthermore, there is also other 14 residues such as Tyr70, Trp84, Trp 432, Tyr121 among others, located primarily in loops between β-strands which contains aromatic rings, and lines a substantial portion of the surface of the gorge. One of them is Trp84 that makes contact with the indole ring and the choline moiety of the neurotransmitter acetylcholinesterase. Indeed, in our docking results it can be seen that the indole ring of Trp84 perfom a π-π interaction with the phenol ring at position 2- of the 4*H*-chromen-4-one framework of kaempferol-3-*O*-galactoside, which could play a key role for the inhibitory activity.

**3. Antiprotozoal evaluation**

**Table S2.** Antiprotozoal results of *Artemisia copa* infusions.

| Specie | *Trypanosoma cruzi*  LD_50_ (µg/mL) | *Leishmania amazonensis*  LD_50_ (µg/mL) |
| --- | --- | --- |
| *Artemisia.copa* Phil. | 131.8 | 457.3 |

All experiments were performed in triplicate (*n* = 3).


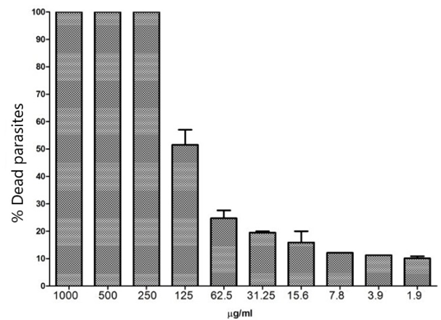

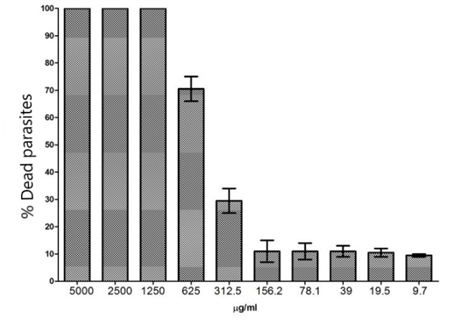


**Figure S3**. Antiprotozoal activity of *Artemisia copa* .Infusion from *Artemisia copa* Phil. (Asteraceae) evaluated against *Trypanosoma cruzi* (left) and *Leishmania amazonensis* (right). Strains were incubated 48h and activity was evaluated and the LD_50_ was determined. Experiments were performed in triplicate and results are expressed as media + SD.

**References**

Adamo, C., and Barone, V. (1999). Toward reliable density functional methods without adjustable parameters: The PBE0 model. *J. Chem. Phys.* 110, 6158–6170. doi:10.1063/1.478522.

Barrientos, R., Fernández-Galleguillos, C., Pastene, E., Simirgiotis, M., Romero-Parra, J., Ahmed, S., et al. (2020). Metabolomic Analysis, Fast Isolation of Phenolic Compounds, and Evaluation of Biological Activities of the Bark From Weinmannia trichosperma Cav. (Cunoniaceae). *Front. Pharmacol.* 11. doi:10.3389/fphar.2020.00780.

Frisch, A. (2009). No Title.

Greenblatt, H. M., Kryger, G., Lewis, T., Silman, I., and Sussman, J. L. (1999). Structure of acetylcholinesterase complexed with (-)-galanthamine at 2.3 Å resolution. *FEBS Lett.* 463, 321–326. doi:10.1016/S0014-5793(99)01637-3.

Levitt, M., and Chothia, C. (1976). Structural patterns in globular proteins. *Nature* 261, 552–558. doi:10.1038/261552a0.

Morris, G. M., Ruth, H., Lindstrom, W., Sanner, M. F., Belew, R. K., Goodsell, D. S., et al. (2009). Software news and updates AutoDock4 and AutoDockTools4: Automated docking with selective receptor flexibility. *J. Comput. Chem.* 30, 2785–2791. doi:10.1002/jcc.21256.

Nachon, F., Carletti, E., Ronco, C., Trovaslet, M., Nicolet, Y., Jean, L., et al. (2013). Crystal structures of human cholinesterases in complex with huprine W and tacrine: Elements of specificity for anti-Alzheimer’s drugs targeting acetyl- and butyryl-cholinesterase. *Biochem. J.* 453, 393–399. doi:10.1042/BJ20130013.

Petersson, G. A., Bennett, A., Tensfeldt, T. G., Al-Laham, M. A., Shirley, W. A., and Mantzaris, J. (1988). A complete basis set model chemistry. I. The total energies of closed-shell atoms and hydrides of the first-row elements. *J. Chem. Phys.* 89, 2193–2218. doi:10.1063/1.455064.

Re, R., Pellegrinia, N., Proteggente, A., Pannalaa, A., Yang, M., and Rice-Evans, C. (1999). Antioxidant activity applying an improved ABTS radical cation decolorization assay. *Free Radic. Biol. Med.* 26, 1231–1237. doi:https://doi.org/10.1016/S0891-5849(98)00315-3.

Silman, I., and Futerman, A. H. (1987). Modes of attachment of acetylcholinesterase to the surface membrane. *Eur. J. Biochem.* 170, 11–22. doi:10.1111/j.1432-1033.1987.tb13662.x.

Sussman, J. L., Harel, M., Frolow, F., Oefner, C., Goldman, A., Toker, L., et al. (1991). Atomic structure of acetylcholinesterase from Torpedo californica: A prototypic acetylcholine-binding protein. *Science (80-. ).* 253, 872–879. doi:10.1126/science.1678899.

Tallini, L. R., Bastida, J., Cortes, N., Osorio, E. H., Theoduloz, C., and Schmeda-Hirschmann, G. (2018). Cholinesterase inhibition activity, alkaloid profiling and molecular docking of chilean rhodophiala (Amaryllidaceae). *Molecules* 23. doi:10.3390/molecules23071532.

Thomsen, R., and Christensen, M. H. (2006). MolDock: A new technique for high-accuracy molecular docking. *J. Med. Chem.* 49, 3315–3321. doi:10.1021/jm051197e.
